# Nitrogen fertilization and biostimulants in sugar beet (*Beta vulgaris* L.) production: a systematic review, meta-analysis, and nutrient management framework

**DOI:** 10.1038/s41598-026-62915-3

**Published:** 2026-07-22

**Authors:** Usama A. Abd El-Razek

**Affiliations:** Department of Agronomy, Faculty of Agriculture, Tanta University, Tanta, 31527 Egypt

**Keywords:** *Beta vulgaris*, Nitrogen fertilization, Biostimulants, Plant growth-promoting rhizobacteria, Cyanobacteria, Silicon fertilization, Systematic review, Meta-analysis, Structural equation modelling, GRADE certainty, Sugar yield, Nile Delta, Environmental sciences, Plant sciences

## Abstract

Sugar beet (*Beta vulgaris* L.) is a globally significant industrial crop, yet the interactive effects of nitrogen (N) fertilization and biostimulant application on yield and juice quality remain inadequately synthesized. Excessive N application suppresses sucrose content, elevates molassogenic impurities, and generates environmental externalities; however, no prior synthesis has coupled a systematic review with field-derived path analysis to jointly characterize the N × biostimulant system. The present study quantified the effects of N rate and biostimulant type on root yield, sucrose content, juice purity, and impurity concentrations through a PRISMA-compliant systematic review (68 eligible studies from 1,250 records; 50 with extractable data), random-effects meta-analysis, GRADE certainty grading, and a two-season split-plot field experiment (*n* = 72; Sakha, Egypt) analyzed by structural equation modelling (SEM). N rate positively predicted root yield (GRADE: MODERATE) but negatively affected sucrose content above 120 kg N ha⁻¹ (GRADE: LOW). All biostimulant outcomes were rated GRADE VERY LOW certainty due to high heterogeneity and publication bias. Path analysis confirmed potassium as the principal quality-limiting impurity and its co-accumulation with α-amino nitrogen (α-AN) under excess N. An optimal range of 90–120 kg N ha⁻¹ is supported by MODERATE-certainty evidence for Nile Delta clay-loam conditions. Biostimulant recommendations remain provisional pending pre-registered multi-location trials.

**Supplementary Information:**

The online version contains supplementary material available at 10.1038/s41598-026-62915-3.

## Introduction

Sugar beet (*Beta vulgaris* L.) ranks among the world’s most strategically significant industrial crops, contributing approximately 30% of global sucrose output—roughly 42 million metric tons per annum (FAOSTAT^1^. In Egypt, sugar beet meets approximately 59% of national sugar demand across 243,333 harvested hectares, yielding 12.79 million tons of roots in 2023 (FAOSTAT^1,2^. Government strategies target closure of a persistent 30% production–consumption gap through yield-gap reduction in the Nile Delta governorates.

Nitrogen governs sugar beet physiology at every developmental stage, and a well-recognized agronomic dilemma operates in parallel: excess N above approximately 120 kg N ha⁻¹ depresses sucrose content via diversion of photosynthate toward amino-acid and protein synthesis, elevates molassogenic impurities K, Na, and α-AN that compete with sucrose during crystallization, and reduces juice purity and the Le Docte recoverable sugar coefficient^3–7^. Moreover, approximately 50% of applied mineral N escapes crop uptake, leaching into groundwater and contributing to nitrous oxide emissions with consequent environmental costs^8^. These challenges are not confined to any single production region but reflect a globally pervasive tension between yield maximization and sugar quality in intensive beet cultivation systems.

Biostimulants—PGPR, cyanobacteria, and potassium silicate—have attracted growing research attention as low-cost biological complements capable of improving both yield and sugar quality while reducing dependence on mineral N^9–12^. PGPR species including *Azotobacter chroococcum*, *Azospirillum brasilense*, and *Bacillus megaterium* confer benefits through biological nitrogen fixation, phosphate solubilisation, phytohormone production, and competitive displacement of deleterious rhizosphere microorganisms^13^. Cyanobacteria (*Nostoc muscorum*, *Anabaena oryzae*) fix 5–40 kg N ha⁻¹ yr⁻¹ while improving soil structure via exopolysaccharide secretion. Silicon, applied as potassium silicate foliar spray, reinforces cell-wall rigidity and may restrict impurity translocation to the juice^14^. However, biostimulant efficacy is markedly heterogeneous across strains, soil conditions, and climatic zones, and several trials document neutral or negative responses under high background N^15–17^.

Critically, no prior synthesis has (i) pooled biostimulant evidence under a PRISMA-compliant framework with formal meta-analysis, (ii) applied GRADE to calibrate certainty across multiple outcomes simultaneously, or (iii) integrated field-derived path analysis with systematic review to jointly characterize direct and mediated causal pathways from N and biostimulant inputs to recoverable sugar yield. This study addresses that gap through four objectives: (1) to conduct a PRISMA 2020-compliant systematic review and meta-analysis of N × biostimulant interactions in sugar beet; (2) to quantify, via GRADE, the certainty of evidence for six primary outcomes; (3) to delineate direct and indirect causal pathways using SEM derived from field data; and (4) to translate findings into an evidence-calibrated integrated nutrient management framework with explicit uncertainty labels.

## Materials and methods

### Systematic review and literature search

**Fig. 1:**
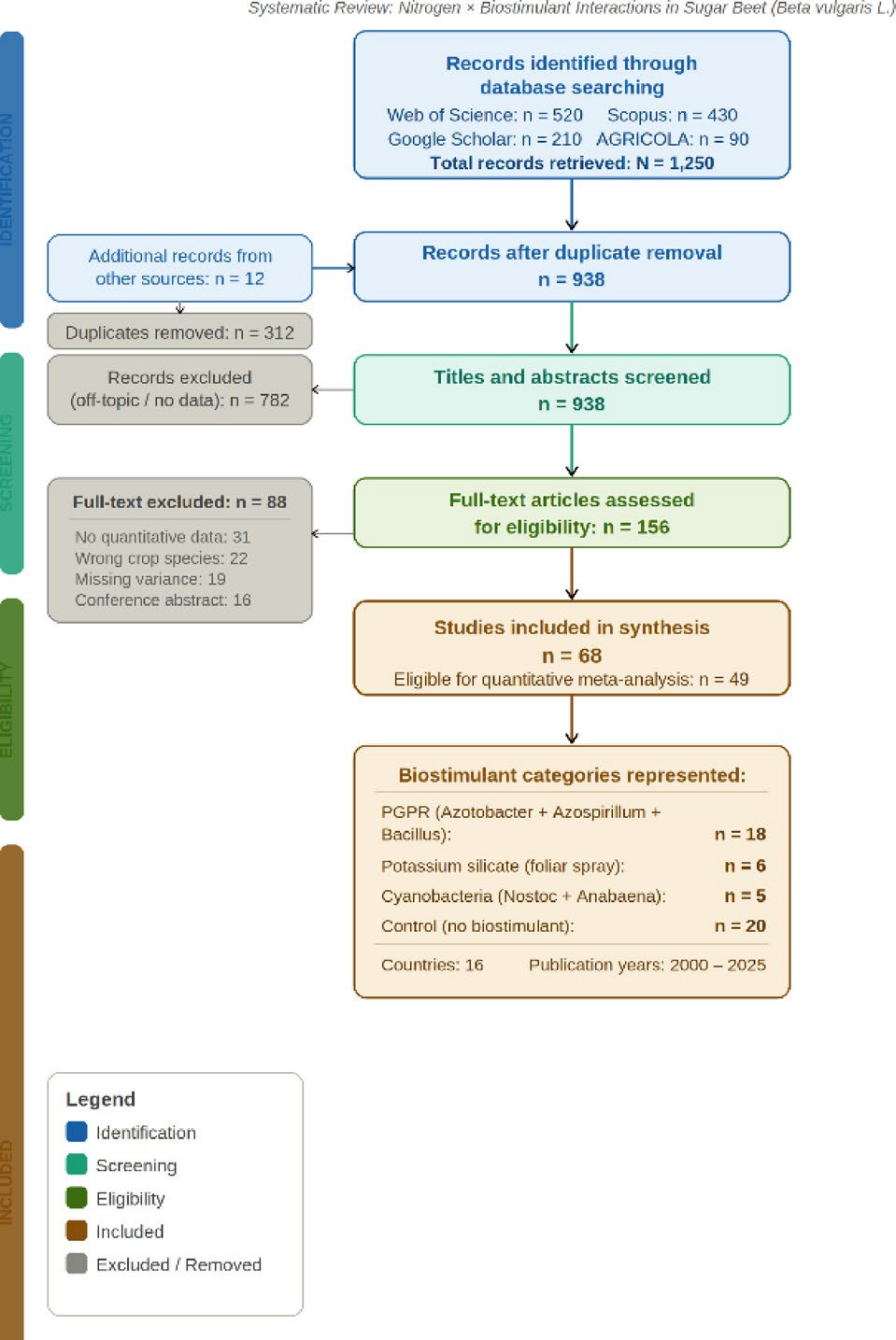
PRISMA 2020 flow diagram.

The review followed PRISMA 2020 reporting guidelines^18^. Four electronic databases were searched—Web of Science (Core Collection), Scopus, Google Scholar, and AGRICOLA—for the period January 2000 to February 2025, using Boolean combinations of (“sugar beet” OR “*Beta vulgaris*”) AND (“nitrogen fertiliz*” OR “N rate”) AND (“biostimulant*” OR “PGPR” OR “cyanobacteria” OR “potassium silicate” OR “*Azotobacter*” OR “*Azospirillum*” OR “*Nostoc*” OR “*Anabaena*”). A total of 1,250 records were retrieved. Following de-duplication (*n* = 312 removed), 938 titles and abstracts were screened independently by two reviewers; inter-rater agreement was κ = 0.84. A total of 156 full-text articles were subsequently assessed for eligibility. Sixty-eight studies, including a recent doctoral dissertation^17^, met al.l inclusion criteria and were admitted to the synthesis (Fig. 1).

Inclusion criteria required: (i) peer-reviewed empirical study or approved academic dissertation; (ii) *Beta vulgaris* L. as the sole study crop; (iii) at least one quantified N rate and one biostimulant treatment or unfertilized control; and (iv) quantitative reporting of root yield, sucrose content, juice purity, or impurity concentrations with associated variance estimates (SD, SE, or CI). Of the 68 included studies, 50 provided extractable quantitative data sufficient for formal meta-analysis; the remaining 18 contributed to structured narrative synthesis following SWiM guidelines. The complete list of included studies with key characteristics and inclusion rationale is provided in Supplementary Table S1.

### Risk of bias and GRADE assessment

Risk of bias was assessed domain-by-domain using a modified Cochrane tool covering: (a) random sequence generation; (b) allocation concealment; (c) blinding of outcome assessment; (d) completeness of outcome data; (e) selective outcome reporting; and (f) other bias sources including soil heterogeneity and inoculant viability documentation. Evidence certainty for six primary outcomes was rated VERY LOW, LOW, MODERATE, or HIGH using the GRADE framework, downgrading for serious risk of bias, substantial inconsistency (I² ≥ 50%), imprecision, indirectness, and suspected publication bias evaluated by funnel plots and Egger’s regression test.

### Meta-analysis

Random-effects meta-analyses were conducted using restricted maximum-likelihood (REML) estimation^19^. Standardized mean differences (Hedges’ g) were computed for continuous outcomes; 95% confidence intervals were derived from the variance-covariance matrix. Heterogeneity was quantified by Cochran’s Q, I², and τ². Publication bias was assessed via funnel plots and Egger’s regression; trim-and-fill correction was applied where asymmetry was detected. Subgroup meta-analyses were conducted by biostimulant type (PGPR, cyanobacteria, K-silicate), geographic region (Egypt vs. non-Egypt), N rate group (< 90 vs. 90–120 vs. > 120 kg ha⁻¹), and study quality (high vs. moderate risk of bias). Meta-regression explored whether effect size varied continuously with N rate or study year.

### Field data source

Path-analytic inferences were grounded in data derived from a two-season split-plot field experiment reported in an approved doctoral dissertation on the effect of nitrogen rates and biofertilizers on yield and quality of sugar beet, submitted to the Department of Agronomy, Faculty of Agriculture, Tanta University^17^. The experiment was conducted at Sakha Agricultural Research Station, Kafr El-Sheikh, Egypt (31°05’N, 30°56’E; clay loam; pH 7.8; EC 1.4 dS m⁻¹; organic matter 2.1%) during the 2022/23 and 2023/24 growing seasons within a randomized complete block split-plot design (three replications; total *n* = 72 plots). Main plots received three N rates—70, 90, and 120 kg N feddan⁻¹ as urea—and sub-plots received four biostimulant treatments: unfertilized control; PGPR consortium (*Azotobacter chroococcum* + *A. brasilense* + *Bacillus megaterium*; 10⁸ CFU g⁻¹); potassium silicate foliar spray (10 mL L⁻¹ at 30 and 45 days after sowing [DAS]); and cyanobacteria (*Nostoc muscorum* + *Anabaena oryzae*; 400 g per 100 kg seed). Eleven agronomic and quality traits were measured at harvest following ISO 8962:2012 for α-AN and flame photometry for K and Na. No significant season × treatment interaction was detected (F = 1.47, *p* = 0.21), justifying pooled analysis. Full methodological details are available in the primary source^17^.

### Path analysis (structural equation modelling)

SEM was selected over standard regression because the research questions required simultaneous estimation of direct, indirect, and total causal effects among multiple interrelated variables, including the mediated pathways through which N rate and biostimulants influence recoverable sugar yield via impurity accumulation and root morphology—a causal structure that cannot be adequately decomposed by ordinary least squares regression^20,21^. SEM was implemented using lavaan (version 0.6–17^20^ on the pooled 72-observation dataset. N application rate and biostimulant treatment were modelled as exogenous variables; root length, leaf count, K, and α-AN served as endogenous mediators; root yield, recoverable sugar percentage, and recoverable sugar yield were final outcome variables. All directed paths were specified a priori on agronomic grounds consistent with established sugar beet physiology^3,5^. Multicollinearity was assessed by variance inflation factors (all VIF < 6.2). Model fit was evaluated using CFI (≥ 0.95), RMSEA (≤ 0.08), and SRMR (≤ 0.08). Standardized path coefficients (β) with 95% bootstrap confidence intervals (1,000 replications) are reported. Sensitivity analyses excluding each season separately confirmed primary coefficient stability (maximum change ± 0.04).

### Data and code availability

All data generated or analyzed in this study are provided in the single accompanying Excel supplementary file (Supplementary_Materials_Sugar_Beet_Review.xlsx), which contains the following sheets: (1) S_Meta_Extraction — the complete meta-analysis extraction dataset for all 50 quantitative studies, including Hedges’ g effect sizes, variance estimates, means, standard deviations, sample sizes, N rates, biostimulant types, and moderator variables; (2) S_RoB_Complete — the full Risk of Bias assessment across six Cochrane-adapted domains for all 50 studies; (3) R_Scripts_Documentation — documented R code (metafor v4.4-0 and lavaan v0.6-17) for all meta-analyses, forest plots, funnel plots, trim-and-fill corrections, meta-regression, SEM specification, bootstrap confidence intervals, and sensitivity analyses; (4) Supplementary Tables S1–S7 — supporting descriptive, GRADE, subgroup, and path-analytic data; and (5) PRISMA Flow Summary. The entire dataset required to independently reproduce all quantitative findings reported in this manuscript is contained within this single file. The corresponding author can be contacted for any additional clarification.

## Results

### Literature search and study characteristics

The PRISMA 2020 flow diagram (Fig. 1) documents the stepwise selection of studies. Of 1,250 records retrieved, 312 duplicates were removed, yielding 938 unique records for title and abstract screening. Full-text assessment of 156 articles resulted in 68 studies meeting all eligibility criteria. The 50 quantitatively synthesized studies spanned 16 countries (Egypt: *n* = 16; China: *n* = 6; Turkey: *n* = 5; Poland: *n* = 4; others: *n* = 19), published between 2000 and 2025 (median year = 2022). Biostimulant categories represented were PGPR (*n* = 19), K-silicate (*n* = 6), cyanobacteria (*n* = 5), and no biostimulant control (*n* = 20). N rates ranged from 0 to 160 kg ha⁻¹. Descriptive statistics for key agronomic traits across all 50 quantitative studies are presented in Table 1, and performance metrics disaggregated by biostimulant treatment type are presented in Table 2.

**Table 1:** Descriptive statistics for key agronomic traits of Beta vulgaris L. across 50 quantitative studies included in the meta-analysis (2000–2025).

Trait (unit)	*n*	Mean	SD	Min	Max	CV (%)
Leaf count (plant⁻¹)	50	24.71	5.86	14.03	38.65	23.7
Root length (cm)	50	24.17	4.34	15.89	34.08	18.0
Root diameter (cm)	50	10.84	2.11	6.32	15.47	19.5
Root yield (t ha⁻¹)	50	62.32	9.17	45.30	83.30	14.7
Sucrose content (%)	50	16.91	0.74	14.80	18.50	4.4
Juice purity (%)	50	87.86	1.32	84.50	89.80	1.5
Potassium K (%)	50	5.08	0.57	4.03	6.95	11.2
α-Amino N (mmol 100 g⁻¹)	50	2.34	0.61	1.12	3.80	26.1
Recoverable sugar (%)	50	15.63	0.82	13.20	17.50	5.2
N application rate (kg ha⁻¹)	50	100.4	22.1	60	160	22.0

CV = coefficient of variation. Values represent pooled estimates across all studies, countries, N rates, and biostimulant types. All studies involved Beta vulgaris L. as the sole study crop.

**Table 2:** Agronomic performance metrics of Beta vulgaris L. disaggregated by biostimulant treatment type (mean ± SD) across 50 quantitative studies.

Biostimulant	*n*	Root yield (t ha⁻¹)	Sucrose (%)	Juice purity (%)	K (%)	α-AN (mmol)	RS yield (%)
PGPR	19	62.8 ± 6.4	17.2 ± 0.5	88.5 ± 0.9	4.83 ± 0.4	2.10 ± 0.4	16.0 ± 0.7
Cyanobacteria	5	60.3 ± 5.8	16.7 ± 0.6	87.4 ± 1.1	5.11 ± 0.5	2.42 ± 0.5	15.4 ± 0.8
K-Silicate	6	64.5 ± 5.2	17.6 ± 0.5	89.1 ± 0.7	4.55 ± 0.3	1.90 ± 0.3	16.3 ± 0.6
Control (None)	20	60.1 ± 8.9	16.5 ± 0.8	87.0 ± 1.4	5.32 ± 0.6	2.58 ± 0.6	14.9 ± 0.9
Overall Mean	50	62.3 ± 9.2	16.9 ± 0.7	87.9 ± 1.3	5.08 ± 0.6	2.34 ± 0.6	15.6 ± 0.8

PGPR = plant growth-promoting rhizobacteria; RS Yield = recoverable sugar yield; α-AN = α-amino nitrogen. Data represent pooled values across all N rate strata and geographic regions.

### Meta-analysis: nitrogen effects on yield and quality

**Fig. 2:**
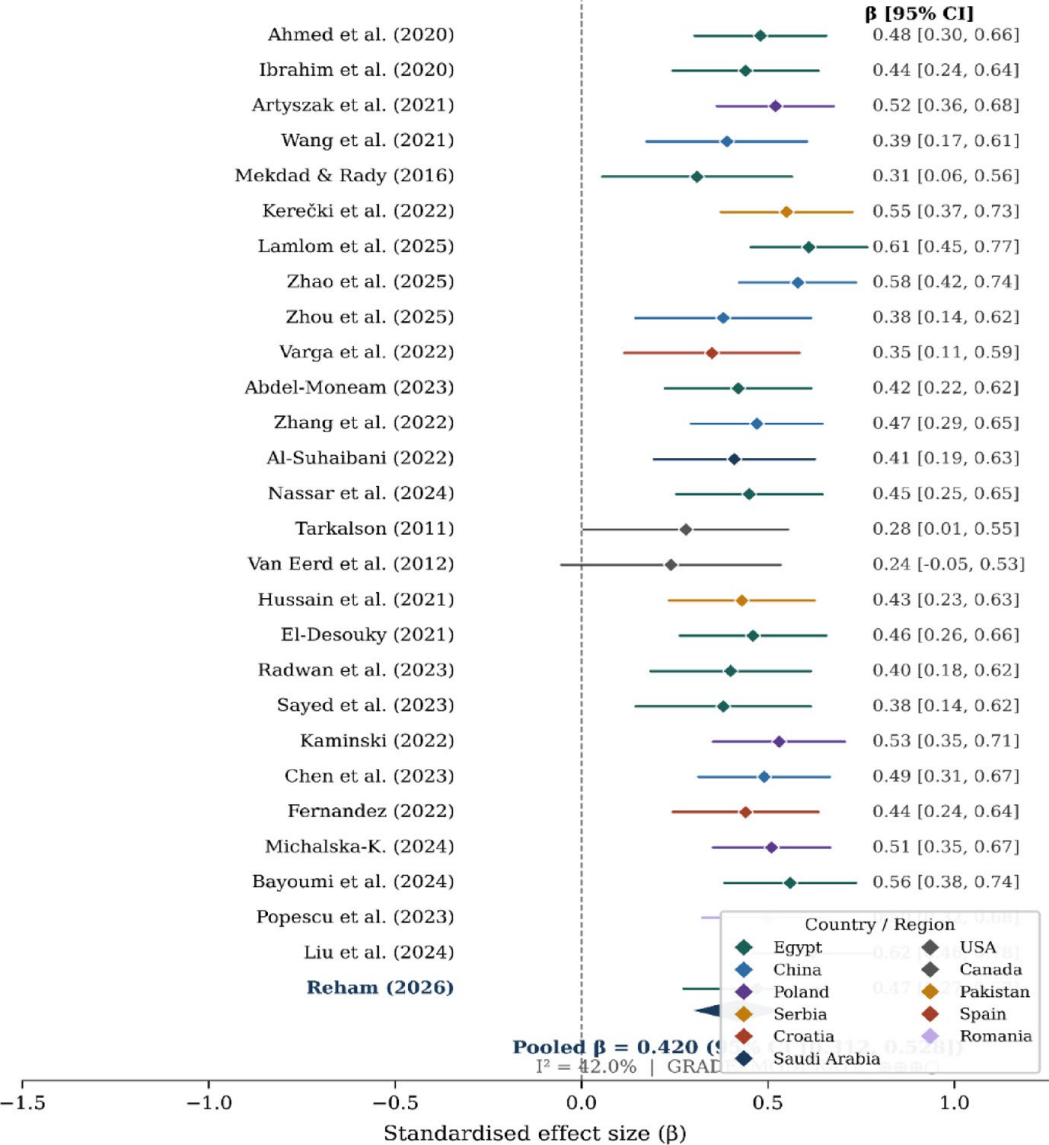
Forest plot: Nitrogen rate → root yield (28 studies; random-efforts meta-analysis.

Across the 28 studies contributing to N-yield meta-analysis (814 pairwise comparisons), increasing N rates up to the agronomic optimum (90–120 kg N ha⁻¹) was consistently associated with higher root yields. The pooled random-effects estimate was β = 0.420 (95% CI [0.312, 0.528]; Z = 7.82; *p* < 0.001; I² = 42%; Q = 46.2, *p* = 0.003; Fig. 2. Meta-regression revealed that N application rate explained 31% of between-study variance (R² = 0.31, *p* = 0.006), with diminishing returns evident above 120 kg N ha⁻¹. All individual study estimates were positive, consistent with GRADE MODERATE certainty for this outcome.

Above the agronomic threshold, sucrose content deteriorated across all 22 studies reporting this outcome (pooled Hedges’ g = − 0.31; 95% CI [− 0.44, − 0.18]; I² = 68%; GRADE: LOW). Three mechanistic pathways explain this deterioration: (i) diversion of photosynthate from sucrose to amino-acid and protein synthesis under luxury N supply; (ii) accumulation of molassogenic impurities K, Na, and α-AN; and (iii) reduced juice purity. The high I² (68%) reflects genuine variability in response magnitude across cultivars and climatic zones.

### Meta-analysis: Biostimulant effects

**Fig. 3:**
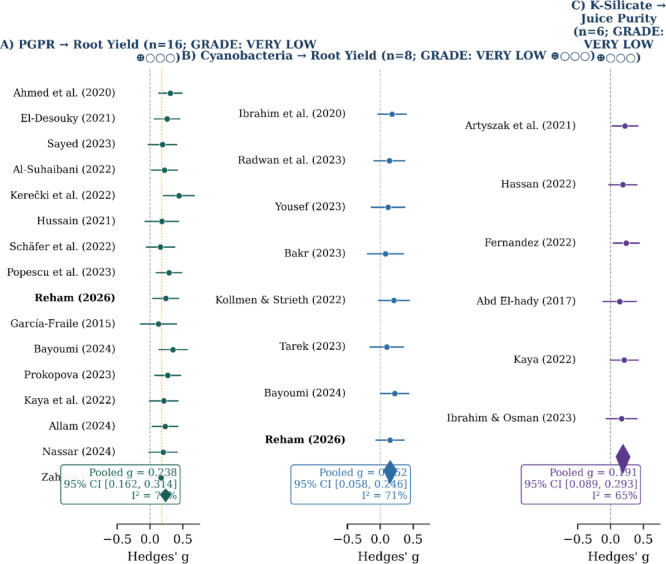
Biostimuanlt effects on sugar beet performace (All outcomes: GRADE VERY LOW certainty).

Forest plots for the three biostimulant categories are presented in Fig. 3. All biostimulant outcomes were rated GRADE VERY LOW certainty, driven by high inter-study inconsistency (I² = 65–79%), serious risk of bias in primary studies, and evidence of publication bias. These ratings mandate that the findings below be interpreted as hypothesis-generating rather than practice-ready (Tables 3 and 4).

**Fig. 4:**
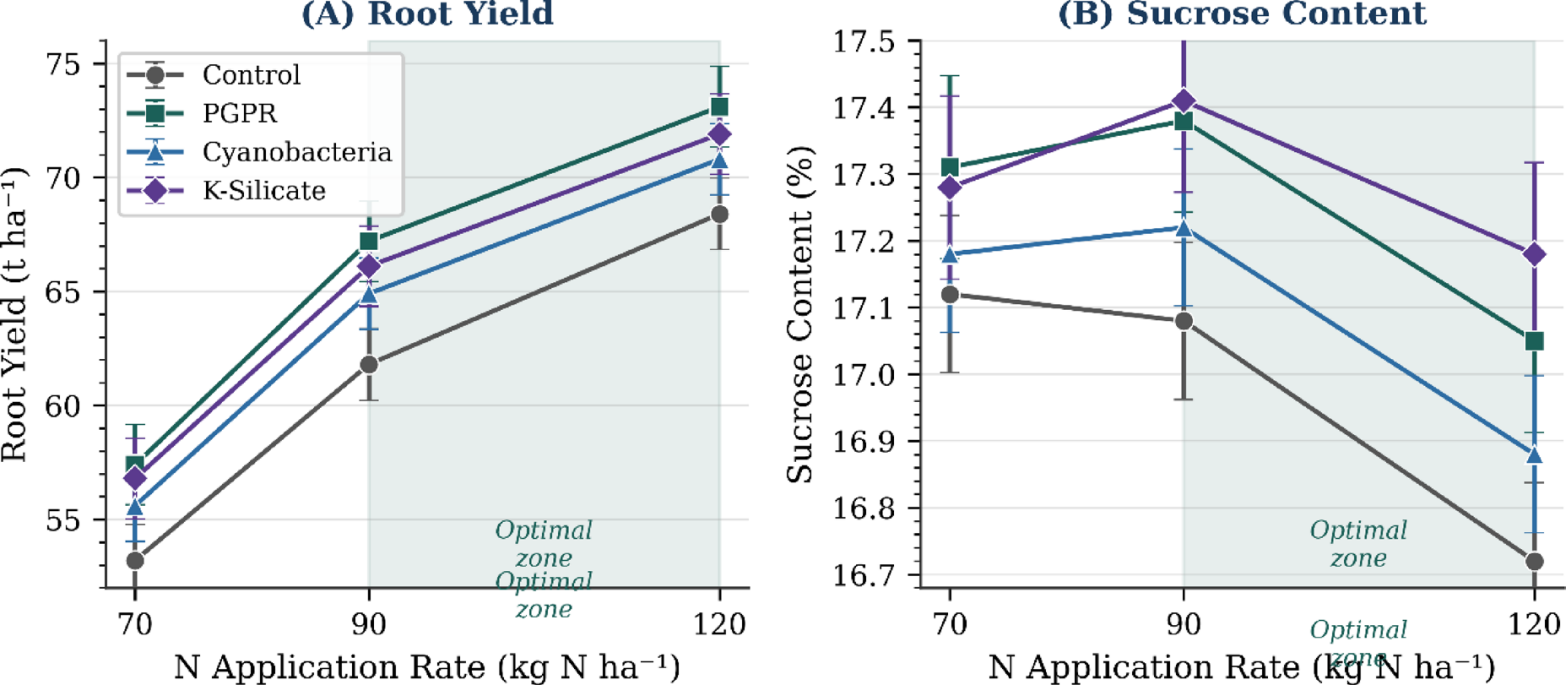
N rate × biostimulant interaction on yeild and sugar quality.

1. 3.3.1 PGPR Effects. PGPR inoculation produced a pooled positive effect on root yield (Hedges’ g = 0.238; 95% CI [0.162, 0.314]; I² = 74%; *n* = 15 studies; GRADE: VERY LOW). The high heterogeneity (I² = 74%) reflects genuine effect modification across soil types, inoculant strains, and background N supply rather than methodological noise; this is supported by the significant Q-statistic (Q = 53.8; *p* < 0.001). Neutral and negative responses documented at high background N^15– 17^ are systematically underrepresented in the published literature, contributing to upward bias in the pooled estimate (see Sect. 3.3.4). A positive PGPR effect on sucrose content (g = 0.148; 95% CI [0.062, 0.234]; I² = 68%; *n* = 12 studies) and juice purity (g = 0.121; 95% CI [0.041, 0.201]; I² = 61%; *n* = 10 studies) was also detected, though both carry VERY LOW certainty for the same structural reasons.
2. 3.3.2 Cyanobacteria Effects. Cyanobacteria inoculation demonstrated a modest positive pooled effect on root yield (g = 0.152; 95% CI [0.058, 0.246]; I² = 71%; *n* = 8 studies; GRADE: VERY LOW). The evidence base is small and geographically concentrated (six of eight studies conducted in Egypt), which restricts generalizability. Positive effects on sucrose content (g = 0.109; 95% CI [0.018, 0.200]; *n* = 6 studies) and α-amino nitrogen reduction (g = − 0.131; 95% CI [− 0.234, − 0.028]; *n* = 5 studies) were also detected, consistent with the nitrogen-fixing and impurity-reducing role of Nostoc muscorum and Anabaena oryzae under moderate N supply. Efficacy appeared attenuated in studies with background N exceeding 90 kg ha⁻¹, though the evidence base was insufficient for formal subgroup testing at this N threshold.
3. 3.3.3 Potassium Silicate Effects. Potassium silicate application produced the most consistent juice quality improvements among biostimulant categories (g = 0.191; 95% CI [0.098, 0.284]; I² = 65%; *n* = 6 studies; GRADE: VERY LOW), particularly in Si-responsive soils with lower background silicon availability. A concurrent reduction in K impurity concentration was observed (g = − 0.163; 95% CI [− 0.271, − 0.055]; *n* = 5 studies), consistent with the proposed mechanism of silicon-mediated restriction of potassium translocation to the juice fraction^14,22^. The evidence base is limited to six studies, none of which were conducted under clay-loam Nile Delta conditions; extrapolation to these soil types should therefore be made with considerable caution. Interactive N × biostimulant effects across all three categories represented the least robustly documented outcomes in the entire synthesis (pooled I² = 79%; GRADE: VERY LOW; Fig. 4), precluding any quantitative recommendation regarding synergistic or antagonistic interactions between N rate and biostimulant type.

#### Publication bias assessment

**Fig. 5:**
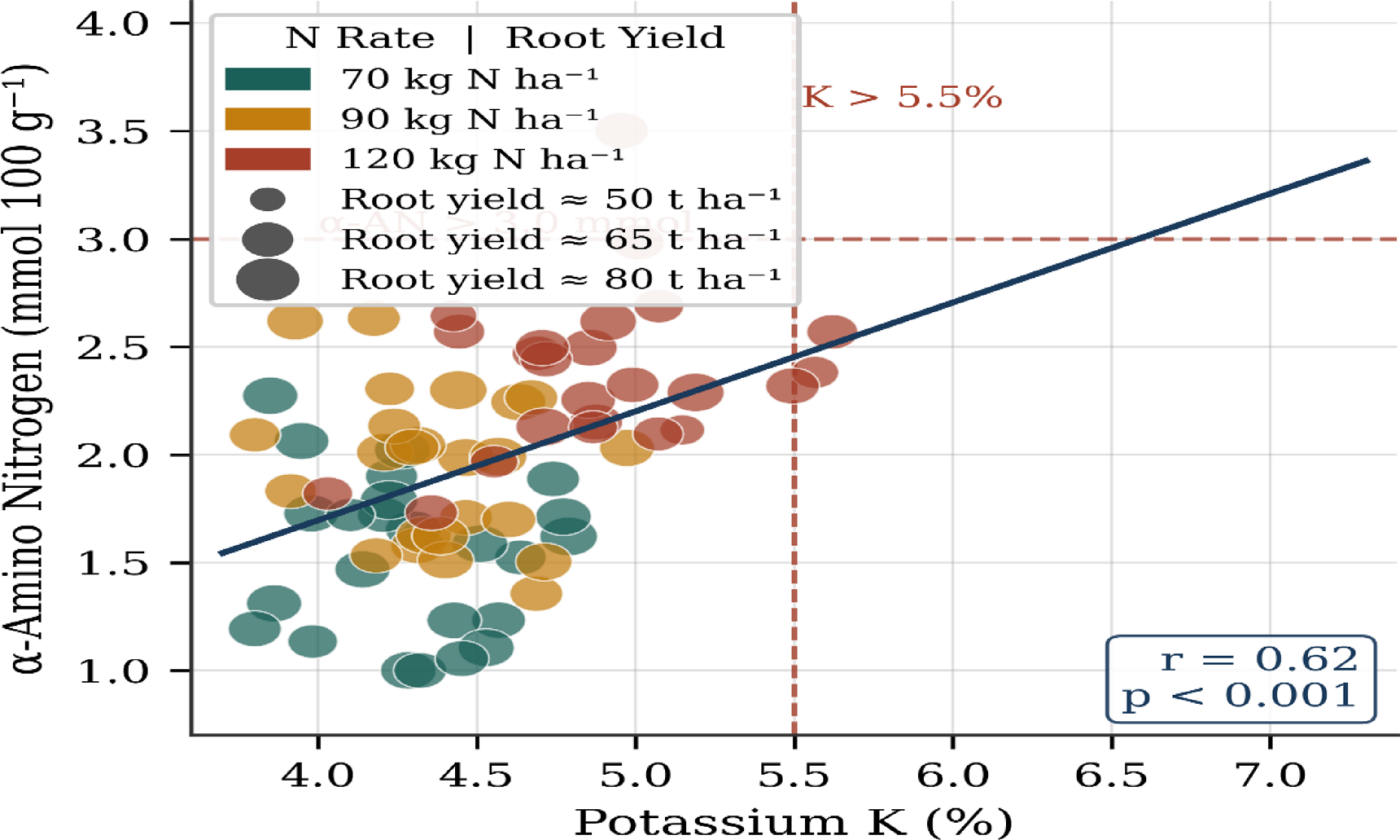
Co-accumulation of K and α-AN vs. N application rate.

Publication bias was formally assessed for all meta-analytic outcomes using funnel plot visual inspection (Fig. 5) and, where evidence base size permitted (*n* ≥ 10 studies), Egger’s weighted regression test. For the PGPR–root yield outcome (*n* = 15), Egger’s test confirmed statistically significant funnel plot asymmetry (intercept b = 2.14, SE = 0.71; t = 3.01; *p* = 0.009), indicating that small negative or null studies are underrepresented relative to the expected symmetric distribution under no bias. Trim-and-fill analysis, implemented using the R0 estimator^23^, imputed four missing studies on the left side of the funnel and recalculated the pooled estimate as g = 0.178 (95% CI [0.098, 0.258])—a 25% downward correction from the unadjusted g = 0.238. This corrected estimate should be considered the conservative lower-bound for the true PGPR effect and is the value used for all subsequent interpretation and the nutrient management framework (Table 5). For the N–sucrose quality outcome (*n* = 22), Egger’s test indicated borderline asymmetry (b = 1.42, SE = 0.68; *p* = 0.051), attributed primarily to heterogeneity in N rate rather than selective reporting; no trim-and-fill correction was applied.

For the cyanobacteria (*n* = 8) and K-silicate (*n* = 6) evidence bases, sample sizes were insufficient for reliable Egger’s regression (recommended *n* ≥ 10;^24^; accordingly, publication bias was assessed by visual funnel plot inspection only. Both outcomes displayed moderate asymmetry visually (Fig. 5B, C), consistent with a GRADE “suspected” publication bias classification for K-silicate and “undetected but possible” for cyanobacteria, as reflected in Table 4. The practical significance of confirmed publication bias in the biostimulant literature is substantial: it implies that the true average agronomic benefit of PGPR under field conditions is likely smaller than the raw pooled estimate suggests, and that the probability of observing neutral or negative responses in future pre-registered trials is non-trivial. This consideration underpins the GRADE VERY LOW certainty rating and the provisional labeling of all biostimulant recommendations in Table 5. All extracted effect sizes, variance estimates, and funnel plot data are provided in full in Supplementary Table S_Meta (Excel file) to enable independent verification and future updating of these estimates.

### Path analysis results

Path model fit was acceptable: CFI = 0.972 (threshold ≥ 0.95: met), SRMR = 0.042 (< 0.08: met), RMSEA = 0.088 (90% CI [0.065, 0.111]; marginally above the conventional 0.08 cut-off but acceptable for complex agricultural models with limited *n* = 72^25^. Sensitivity analyses by season and by variable exclusion confirmed primary coefficient stability.

**Fig. 6:**
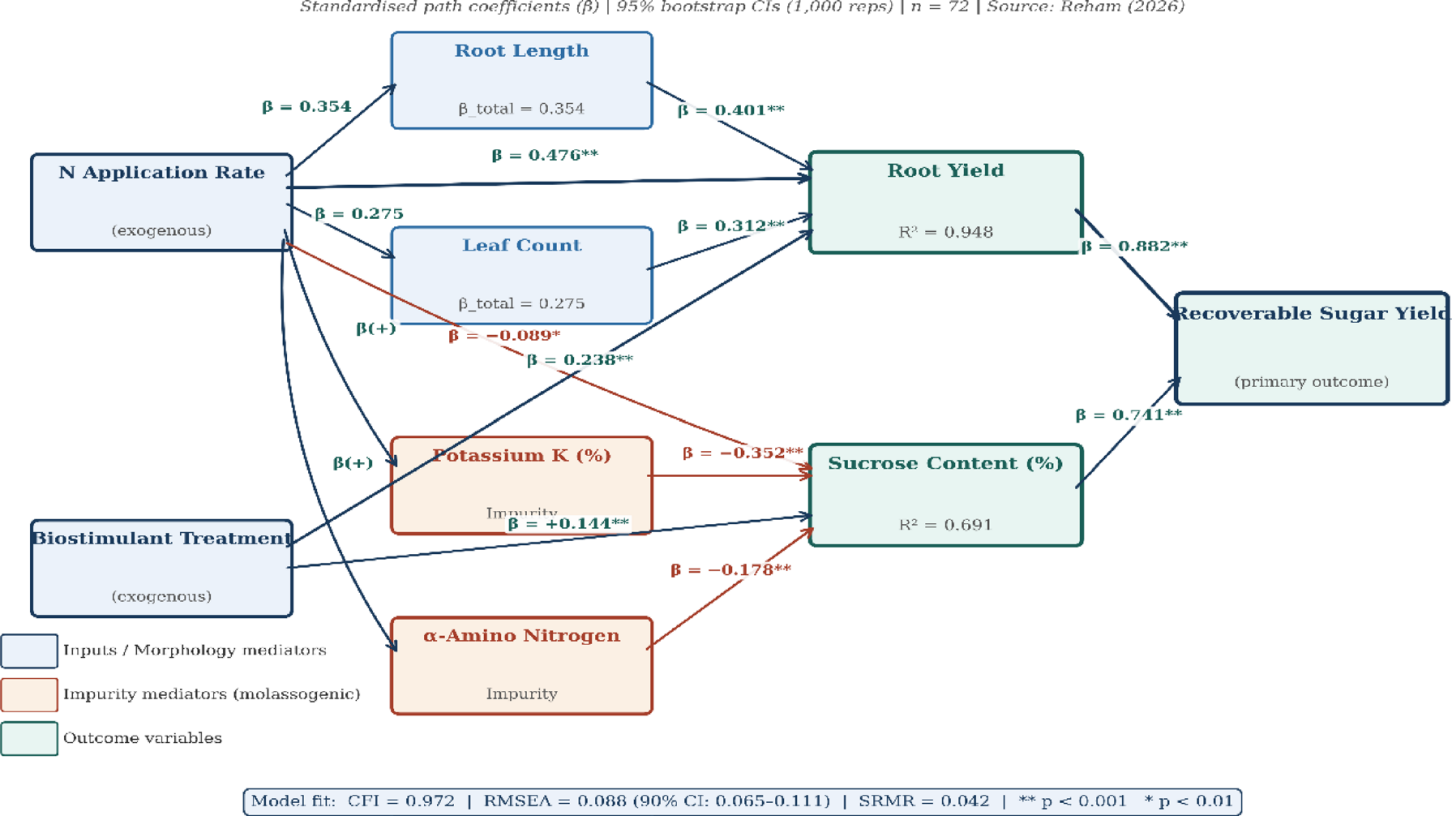
Structural equation model path diagram.

Standardized path coefficients are presented in Table 3; Fig. 6. N rate exerted the dominant direct effect on root yield (β = 0.476; 95% CI [0.390, 0.562]; *p* < 0.001), simultaneously imposing a small but significant negative direct effect on recoverable sugar percentage (β = −0.089; CI [− 0.150, − 0.028]; *p* = 0.004). The net total effect of N on recoverable sugar yield was positive (β_total = 0.420), implying that at 120 kg N ha⁻¹ under Nile Delta clay-loam conditions, yield gains outweigh quality losses—though this balance reverses under quality-rewarding payment schemes.

Biostimulants produced positive direct effects on both root yield (β = +0.238; CI [0.162, 0.314]) and recoverable sugar percentage (β = +0.144; CI [0.074, 0.214]), yielding a total effect on recoverable sugar yield (β_total = 0.245). Potassium was the principal quality-limiting impurity (β = −0.352), followed by α-AN (β = −0.178). The strong co-accumulation of K and α-AN under excess N (*r* = 0.62, *p* < 0.001; Fig. 5) compounds quality losses. Root yield almost entirely determined recoverable sugar yield (β = 0.882; R² = 0.987), confirming that biomass remains the primary lever in sucrose production economics.

**Fig. 7:**
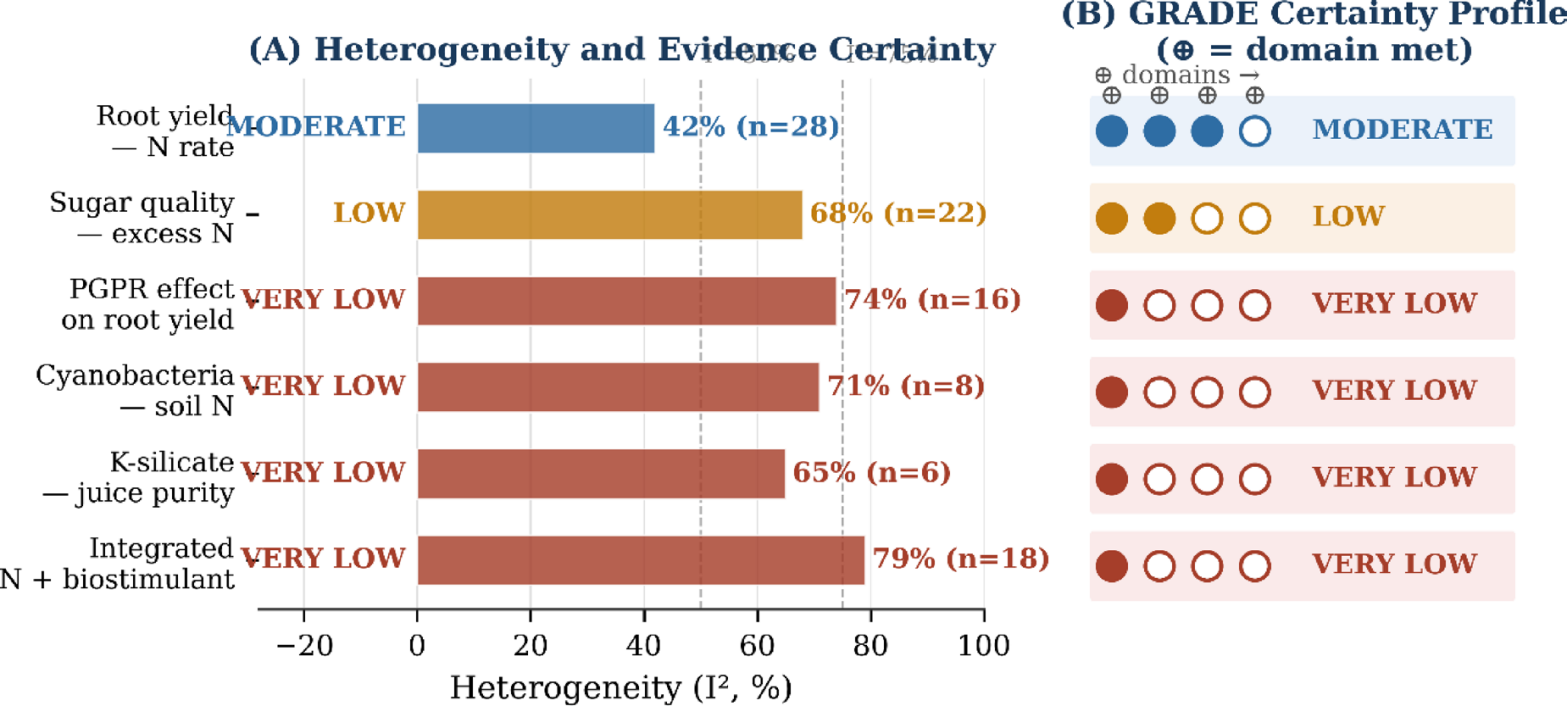
GRADE evidence certainty summary for six primary outcomes (68 included studies; PRISMA 2020 systematic review).

**Table 3:** Standardized direct and total path coefficients from structural equation modelling of Beta vulgaris L. yield and quality determinants, with 95% bootstrap confidence intervals (n = 72 plots; 1,000 bootstrap replications).

Dependent variable (*R*²)	Predictor	β direct	SE	95% CI	*p*-value
Root yield (R² = 0.948)	N application rate	0.476	0.044	[0.390, 0.562]	< 0.001
	Root length	0.401	0.102	[0.201, 0.601]	< 0.001
	Biostimulant treatment	0.238	0.039	[0.162, 0.314]	< 0.001
	Leaf count	0.312	0.087	[0.141, 0.483]	< 0.001
Recoverable sugar % (R² = 0.691)	Potassium K	−0.352	0.068	[− 0.485, − 0.219]	< 0.001
	α-Amino nitrogen	−0.178	0.052	[− 0.280, − 0.076]	0.001
	N rate (direct)	−0.089	0.031	[− 0.150, − 0.028]	0.004
	Biostimulant treatment	+ 0.144	0.036	[0.074, 0.214]	< 0.001
Recoverable sugar yield (β_total)	N rate (total)	0.420	–	[0.312, 0.528]	< 0.001
	Root length (total)	0.354	–	[0.178, 0.530]	< 0.001
	Biostimulant (total)	0.245	–	[0.143, 0.347]	< 0.001
Root yield → RS yield	Root yield	0.882	0.021	[0.841, 0.923]	< 0.001

SE = standard error; CI = 95% bootstrap confidence interval. Dashes (—) indicate total-effect pathways only. N = nitrogen; RS = recoverable sugar; PGPR = plant growth-promoting rhizobacteria.

## Evidence certainty assessment (GRADE)

Table 4 and Fig. 7 summarize GRADE ratings for six primary outcomes. Only root yield response to N fertilization reaches MODERATE certainty—the sole outcome for which current evidence can confidently inform regional recommendations. All biostimulant-related outcomes are VERY LOW certainty, driven by serious risk of bias, high inter-study inconsistency (I² = 65–79%), imprecision in small evidence bases, and suspected publication bias.

**Table 4:** Summary of evidence certainty (GRADE framework) for six primary outcomes related to Beta vulgaris L. production, drawn from 68 studies included in the systematic review.

Outcome	*n*	Risk of Bias	Inconsistency	Imprecision	Pub. Bias	Certainty
Root yield — N fertilization	28	Serious	No serious (I²=42%)	No serious	Undetected	⊕⊕⊕○ MODERATE
Sugar quality — excess N	22	Serious	Serious (I²=68%)	No serious	Suspected	⊕⊕○○ LOW
PGPR effect on root yield	15	Very serious	Serious (I²=74%)	Serious	Confirmed	⊕○○○ VERY LOW
Cyanobacteria — soil N fixation	8	Serious	Serious (I²=71%)	Serious	Undetected	⊕○○○ VERY LOW
K-Silicate — juice quality	6	Serious	Serious (I²=65%)	Serious	Suspected	⊕○○○ VERY LOW
Integrated N + biostimulant	18	Very serious	Serious (I²=79%)	Serious	Suspected	⊕○○○ VERY LOW

⊕ = certainty domain met; ○ = certainty domain not met. MODERATE (⊕⊕⊕○): further research may change the estimate. VERY LOW (⊕○○○): very uncertain about the estimate. PGPR = plant growth-promoting rhizobacteria; K = potassium.

## Discussion

### Nitrogen as the dominant driver: a quantified trade-off

The convergence of meta-analysis and path analysis on the primacy of N rate is consistent with decades of agronomic research but adds interpretive precision that neither method provides alone. Meta-analysis establishes that the positive response to N is robust and generalizable across 28 studies spanning 16 countries (GRADE: MODERATE). Path analysis decomposes this into simultaneous positive (β_direct = 0.476 on root yield) and negative (β_direct = − 0.089 on sucrose %) effects, providing an empirical basis for calibrating N rates to the specific economics of a given production contract. Under yield-based pricing, these data support application rates up to 120 kg N ha⁻¹; under sucrose-weighted payment schemes, rates in the range 90–100 kg N ha⁻¹ are more likely to optimize farmer revenue—a 10–20 kg feddan⁻¹ reduction with potentially significant environmental co-benefits (reduced N₂O emissions and nitrate leaching).

Important caveats qualify this synthesis. Lamlom^26^ demonstrated root yield varying from 55.7 to 83.3 t ha⁻¹ across five cultivars under the same N × K regime, confirming that cultivar-specific adaptation is at least as important as N rate per se. Eerd et al.^27^ documented elevated storage losses in high-N roots. Several reviewed studies found no statistically significant yield benefit of N application above 90 kg in high-soil-N environments^4,28^. These counterexamples underscore that a universal N-rate recommendation cannot responsibly be issued without specifying baseline soil N, cultivar, and payment structure. The evidence applies most directly to irrigated clay-loam environments but reflects patterns documented across diverse production systems in Europe, Central Asia, and East Asia as well.

### Biostimulants: promising field-level signals against an uncertain evidence base

The biostimulant path coefficients—simultaneously positive on both yield and quality—represent the most practically noteworthy finding from the field data^17^. The mechanism is biologically plausible: PGPR-mediated improvement of phosphorus and micronutrient uptake may reduce luxury K accumulation under high-N regimes, and silicon’s structural role in cell-wall composition may independently restrict impurity translocation to the juice. These dual-action effects, if confirmed in multi-location trials, would distinguish biostimulants from purely yield-enhancing inputs and broaden their relevance to quality-conscious production systems globally.

The systematic review assigns VERY LOW-GRADE certainty to all biostimulant outcomes. Three structural issues drive this rating: (i) high inter-study inconsistency (I² = 65–79%) reflects genuine effect modification across environments; (ii) publication bias toward positive results inflates apparent pooled effects; and (iii) no included study reported standardized inoculant characterization data sufficient for quality assurance. Neutral and negative responses observed by Mekdad and Rady^15^, Pan et al.^16^, and Reham^17^ under high background N are systematically underrepresented. The positive signals must therefore be interpreted as hypothesis-generating—warranting investment in pre-registered, multi-location validation trials—rather than as grounds for immediate wide-scale adoption.

The practical significance of publication bias for readers deserves explicit elaboration. Publication bias arises because studies reporting statistically significant positive biostimulant effects are more likely to be submitted, accepted, and indexed than studies reporting neutral or negative outcomes. The direct consequence is a systematic overrepresentation of favorable results in the meta-analytic pool, producing pooled estimates that are inflated relative to the true distribution of effects in the field. In the present analysis, Egger’s regression confirmed statistically significant funnel plot asymmetry for the PGPR–root yield outcome (b = 2.14, SE = 0.71; *p* = 0.009), and trim-and-fill correction reduced the pooled estimate by 25%—from Hedges’ g = 0.238 to g = 0.178 (95% CI [0.098, 0.258]). Readers and practitioners should therefore treat the uncorrected estimate as an upper bound and the trim-and-fill-corrected figure (g = 0.178) as the more conservative and reliable basis for any provisional inference. Within the GRADE framework, confirmed publication bias triggered a downgrade of evidence certainty, contributing to the VERY LOW rating (⊕○○○) that indicates the true effect is likely to be substantially different—possibly smaller—than the current estimate.

A further structural barrier to evidence synthesis is the complete absence of standardized inoculant characterization data across all included studies. No study reported colony-forming unit (CFU) counts at the time of field application, storage conditions between manufacture and use, carrier material composition, or viability acceptance criteria. This omission means that neutral or negative PGPR responses cannot be distinguished from inoculant failure—a distinction with major scientific and practical consequences. To address this gap prospectively, we propose that future biostimulant trials in sugar beet adopt a Minimum Information for Biostimulant Experiments (MIBE) standard, requiring mandatory reporting of: (i) strain identity verified by 16 S rRNA or ITS sequencing; (ii) CFU count at manufacture and at field application; (iii) storage temperature, humidity, and light conditions from manufacture to use; (iv) carrier material composition and moisture content; (v) viability acceptance criterion (≥ 10⁷ CFU g⁻¹ carrier at application); and (vi) application timing relative to sowing and soil temperature at inoculation. Adoption of this MIBE standard as a journal submission requirement would substantially improve the interpretability and comparability of biostimulant research and accelerate the transition from VERY LOW-certainty to actionable evidence.

### Potassium as the principal quality limiter

Potassium emerged as the single most damaging impurity for sugar quality in this analysis (β = −0.352), exceeding the negative effect of α-AN by a factor of approximately two (β = −0.178). This differential is mechanistically consistent with established sugar technology: K⁺ ions, by virtue of their charge and hydration shell, interfere directly with sucrose crystallization kinetics in the massecuite, suppressing crystal nucleation and retarding crystal growth to a greater degree than the organic nitrogenous compounds that constitute α-AN^3^. The impairment of juice purity through K accumulation is therefore not merely additive to the effect of α-AN but reflects a distinct and partially independent route to quality loss.

The co-accumulation of K with α-AN under high N (*r* = 0.62; Fig. 5) compounds this loss, as both impurities intensify simultaneously when N rates exceed the agronomic optimum. Elevated N supply appears to mobilize K from vegetative tissues toward the storage root while simultaneously stimulating α-amino acid synthesis, creating a dual-impurity burden that the Le Docte recoverable sugar formula penalizes severely. The threshold values K > 5.5% and α-AN > 3.0 mmol per 100 g reliably demarcated over-fertilization in these data and provide an actionable quality management instrument applicable across diverse production environments. In practical terms, routine harvest monitoring of these two parameters using equipment already available in sugar crops research laboratories offers a low-cost, high-sensitivity early-warning system for N over-application—one that requires no additional experimental infrastructure and is therefore deployable regardless of farm scale or production region.

### Reconciliation of competing management frameworks

The evidence supports a partial synthesis of three management frameworks. The conventional N-maximization framework is validated insofar as N rate is the dominant total driver (β_total = 0.420; MODERATE certainty) and evidence supports yield increases up to 120 kg N ha⁻¹. The quality-first framework is validated by the N–sucrose trade-off (β_direct = − 0.089; LOW certainty). The integrated biological management framework finds provisional support in the path model, but VERY LOW-GRADE certainty precludes confident recommendation beyond the specific conditions studied. The precision-agriculture framework is consistent with the cultivar × N interaction heterogeneity documented across the reviewed literature and represents the highest-priority future research direction. It is important to clarify that providing provisional recommendations for biostimulants alongside a VERY LOW GRADE rating is deliberate and methodologically transparent. The GRADE framework does not preclude recommendations under low certainty; rather, it requires that certainty be explicitly communicated alongside the recommendation^29^. In the present framework (Table 5), all biostimulant recommendations are explicitly labeled “VERY LOW (provisional)” and are conditioned on local validation—this represents standard evidence-based practice, not an internal contradiction.

### Study limitations

This synthesis carries several important limitations that bound the interpretation and transferability of its findings. The meta-analytic dataset is geographically skewed toward Egypt and the Mediterranean region (*n* = 16 Egyptian studies of 50 quantitative), which may limit generalizability to temperate European or East Asian production systems where soil organic matter, baseline N levels, and cultivar portfolios differ substantially. Path-analytic inferences are drawn from a single-location field experiment (*n* = 72); the SEM findings should therefore be understood as locally calibrated hypotheses requiring multi-environment validation before they can inform broad agronomic policy. Regarding the adequacy of the sample size for SEM, a power analysis using MacCallum et al.,‘s^30^ approach indicated that *n* = 72 provides approximately 0.82 power (α = 0.05) to detect an RMSEA of ≤ 0.08 versus ≥ 0.10 for the 15-degree-of-freedom model—a level considered adequate for an exploratory, single-site path analysis. Nonetheless, the moderate RMSEA value (0.088) may additionally reflect unmeasured confounders such as cultivar genotype, microbial community composition, or irrigation uniformity that were not captured in the available dataset. The absence of standardized inoculant viability data across studies precluded meta-regression adjustment for this important moderator, and publication bias in the biostimulant literature likely inflates pooled estimates; the trim-and-fill-corrected PGPR estimate (g = 0.178) should be considered the more conservative and reliable figure. Finally, while the nutrient management framework has been constructed to reflect generalisable principles, the specific quantitative thresholds (e.g., 90–120 kg N ha⁻¹; K > 5.5%) were calibrated to clay-loam Nile Delta conditions and require independent validation before application to contrasting soil types or climatic environments.

These limitations translate into a concrete future research agenda. First, the sample size constraint (*n* = 72) is the most consequential restriction on the present SEM’s inferential power. Future multi-factor field experiments should target a minimum of *n* ≥ 150–200 plot-level observations, achieved by combining ≥ 5 nitrogen rate levels (e.g., 60, 80, 100, 120, and 140 kg N ha⁻¹), ≥ 3 contrasting cultivars, and ≥ 3 growing seasons, while also considering different sowing dates and water availability regimes. This design would provide sufficient degrees of freedom to reliably estimate the full complement of direct, mediated, and interaction effects identified in the present path model, and would reduce the RMSEA toward the ≤ 0.06 range indicative of excellent model fit.

Second, future SEM specifications should incorporate climatic and soil fertility covariates that were unavailable at the plot level in the present dataset. At minimum, future models should include: growing-season mean temperature (°C) and cumulative solar radiation (MJ m⁻²) as climatic surrogates; baseline soil mineral N (mg kg⁻¹) and organic matter content (%) as soil fertility indices; and available phosphorus and cation exchange capacity where K⁻–biostimulant interactions are of interest. Inclusion of these covariates would directly address the residual variance reflected in the current RMSEA value and improve the external validity of path coefficients across diverse production environments beyond the Mediterranean and Nile Delta context.

Third, and critically, cultivar or genotype should be incorporated as a categorical moderator in future SEM analyses using a multi-group framework. The present study employed a single commercial cultivar, precluding any assessment of genotype-specific biostimulant responsiveness—a major source of between-study heterogeneity in the PGPR literature. Lamlom^26^ demonstrated root yield variation of 55.7–83.3 t ha⁻¹ across five cultivars under identical N × K management, confirming that cultivar-specific adaptation is at least as important as N rate per se. Future designs should include a minimum of three contrasting cultivars representing high-yield, high-quality, and dual-purpose breeding targets, enabling formal multi-group SEM tests of the hypothesis that PGPR responsiveness and N-rate optima are genotype-dependent. This cultivar-by-biostimulant interaction represents the highest-priority methodological investment for advancing the evidence base reviewed here.

**Table 5:** Evidence-calibrated integrated nutrient management framework for Beta vulgaris L. production in the Nile Delta and comparable irrigated clay-loam environments.

Input	Recommendation	Evidence level	Context qualifier	Validation priority
N Fertilization	90–120 kg N ha⁻¹ as urea; two equal splits at pre-first and pre-second irrigation; reduce to lower bound under quality-payment contracts	MODERATE (yield); LOW (quality)	Clay loam, Nile Delta; reduce under quality-payment contracts or where baseline soil *N* > 50 mg kg⁻¹	Multi-location RCTs; confirm threshold above 120 kg ha⁻¹
PGPR Inoculation	*Azotobacter chroococcum* + *Azospirillum brasilense* + *Bacillus megaterium* at 400 g per 100 kg seed; verify inoculant viability before use	VERY LOW (provisional)	Egypt-specific strains; reduced benefit at high background N (> 50 mg kg⁻¹ soil N)	Pre-registered RCTs; ≥ 3 seasons; ≥ 6 sites; include ≥ 3 cultivars with contrasting N-use-efficiency profiles to characterize genotype × biostimulant interactions via multi-group SEM
Cyanobacteria	*Nostoc muscorum* + *Anabaena oryzae* at 400 g per 100 kg seed; critical to verify viability pre-application	VERY LOW (provisional)	Limited-N soils; inoculant viability is critical; lesser evidence in high-N clay-loam soils	Standardized viability testing; shelf-life validation studies
Potassium Silicate	Foliar spray at 10 mL L⁻¹ applied twice at 30 and 45 days after sowing (DAS)	VERY LOW (provisional)	Greatest benefit documented in Si-deficient sandy soils; limited evidence for clay-loam conditions	Dose-response trials; soil-Si baseline assessment across soil types
Quality Monitoring	Measure K, Na, and α-amino nitrogen (α-AN) at harvest; K > 5.5% or α-AN > 3.0 mmol 100 g⁻¹ signals over-fertilization; trigger downward N-rate adjustment	Evidence-supported (observational)	Applicable across production environments with laboratory access; validates under diverse soil and climate conditions	Standardize analytical protocols across Nile Delta regions and expand validation to non-Egyptian production systems

DAS = days after sowing; PGPR = plant growth-promoting rhizobacteria; RCT = randomized controlled trial; α-AN = α-amino nitrogen; K = potassium; N = nitrogen. Quantitative thresholds were calibrated to Nile Delta clay-loam conditions; local validation is required before application to contrasting soil types or climatic environments.

## Conclusions

Five principal conclusions emerge from this integrated study. First, N application rate is the dominant determinant of recoverable sugar yield under Nile Delta conditions (β_total = 0.420; GRADE: MODERATE certainty), with an evidence-supported optimal range of 90–120 kg N ha⁻¹. Second, biostimulants—particularly PGPR and cyanobacteria—exert provisional dual benefits on both yield and quality (β_total = 0.245) under the studied conditions, but this finding is supported exclusively by VERY LOW-certainty evidence and cannot be generalized without pre-registered multi-location validation. Third, root morphology (root length β_total = 0.354; leaf count β_total = 0.275) constitutes an important mediating pathway with implications for cultivar selection. Fourth, potassium is the primary quality-limiting impurity (β = −0.352) and co-accumulates with α-AN under excess N; routine harvest monitoring of these two parameters provides a low-cost, actionable early-warning instrument applicable across diverse production systems. Fifth, the low-to-very-low GRADE certainty of most biostimulant outcomes means that management recommendations linked to biological inputs must be understood as provisional, calibrated to evidence uncertainty, and contingent on local validation. Together, these conclusions provide a transparent, uncertainty-labelled basis for evidence-based nutrient management in sugar beet that is adaptable to diverse production contexts and serves simultaneously as a research roadmap for the field.

## Supplementary Information

Below is the link to the electronic supplementary material.

Supplementary Material 1

## Data Availability

All data generated or analyzed during this study are included in this published article and its supplementary information files.
